# In Vivo Plant Bio-Electrochemical Sensor Using Redox Cycling

**DOI:** 10.3390/bios13020219

**Published:** 2023-02-02

**Authors:** Tali Dotan, Aakash Jog, Kian Kadan-Jamal, Adi Avni, Yosi Shacham-Diamand

**Affiliations:** 1 Department of Physical Electronics, School of Electrical Engineering, Faculty of Engineering, Tel Aviv University, Tel Aviv 69978, Israel; 2 Department of Material Science and Engineering, Faculty of Engineering, Tel Aviv University, Tel Aviv 69978, Israel; 3 School of Plant Sciences and Food Security, Faculty of Life Sciences, Tel Aviv University, Tel Aviv 69978, Israel; 4 TAU/TiET Food Security Center of Excellence (TTFSCoE), Thapar Institute of Engineering and Technology, Patiala 147004, India

**Keywords:** electrochemical biosensing, redox cycling, plant-based functional sensor, heat shock plant sensor, in vivo plant sensors

## Abstract

This work presents an in vivo stem-mounted sensor for *Nicotiana tabacum* plants and an in situ cell suspension sensor for *Solanum lycopersicum* cells. Stem-mounted sensors are mechanically stable and less sensitive to plant and air movements than the previously demonstrated leaf-mounted sensors. Interdigitated-electrode-arrays with a dual working electrode configuration were used with an auxiliary electrode and an Ag/AgCl quasi-reference electrode. Signal amplification by redox cycling is demonstrated for a plant-based sensor responding to enzyme expression induced by different cues in the plants. Functional biosensing is demonstrated, first for constitutive enzyme expression and later, for heat-shock-induced enzyme expression in plants. In the cell suspension with redox cycling, positive detection of the enzyme β-glucuronidase (GUS) was observed within a few minutes after applying the substrate (pNPG, 4-Nitrophenyl β-D-glucopyranoside), following redox reactions of the product (p-nitrophenol (pNP)). It is assumed that the initial reaction is the irreversible reduction of pNP to p-hydroxylaminophenol. Next, it can be either oxidized to p-nitrosophenol or dehydrated and oxidized to aminophenol. Both last reactions are reversible and can be used for redox cycling. The dual-electrode redox-cycling electrochemical signal was an order of magnitude larger than that of conventional single-working electrode transducers. A simple model for the gain is presented, predicting that an even larger gain is possible for sub-micron electrodes. In summary, this work demonstrates, for the first time, a redox cycling-based in vivo plant sensor, where diffusion-based amplification occurs inside a tobacco plant’s tissue. The technique can be applied to other plants as well as to medical and environmental monitoring systems.

## 1. Introduction

Data-driven agriculture (i.e., precision agriculture) is an important technology, addressing the global food security problem. Food security is affected, on the one hand, by the increasing demand due to population increase and rising standards of living and, on the other hand, by the stress on resources (i.e., land, water, etc.) and climate conditions. Global warming and dehydration have a considerable negative effect on crop yields and food security. High temperature, even for short periods, affects crop growth. Abiotic stress has globally contributed to higher yield loss than any other single biotic or abiotic factor [1]. Thus, achieving good agricultural productivity under climate change requires research and development of new methods for precision agriculture. This includes developing better sensors, either for lab use or field deployment, which can generate data for optimizing food production.

Bio-electrochemical plant-based sensors have been demonstrated to be a promising method for early and low-cost detection of stress in plants. Leaf-mounted functional bio-electrochemical sensors have been demonstrated to yield electrochemical currents in the range of nA to a few μA, for field-mounted units [2]. In this manuscript, we present an improved system for plant-based biosensing, compared to a previously published work. The novel system has been studied for sensing and monitoring cellular enzymatic activity that produces electrochemically active species. These studies were performed both in vivo (e.g., plant mounted) and in situ (e.g., cell lines) settings. The improved performance, compared to conventional electrodes, can be attributed to the amplification in a dual-working electrode electrochemical chip where the electrodes are biased for “redox cycling”. This is significant for plant-mounted sensors where the novel stem-mounted approach is more stable and lasts longer, compared to the leaf-mounted units [2].

The sensing paradigm that is presented here includes the expression of the β-glucuronidase (GUS) enzyme in the plant or cell suspension, followed by an enzymatic reaction with a substrate (pNPG, 4-Nitrophenyl β-D-glucopyranoside), releasing an electroactive analyte (*p*-nitrophenol). Electrochemical detection of the *p*-nitrophenol was performed using an interdigitated-electrode array (IDA). The presented work shows the feasibility of detecting GUS using plants and cell lines which constitutively express the enzyme, and then demonstrates a sensing of heat shock using stress-induced expression of the enzyme.

### 1.1. Plant-Based Biosensing

In a typical plant-based functional sensor, the plant’s response is characterized by either changing one of its properties (e.g., color, dielectric properties, impedance, etc.), or by generating a signal via genetic expression of biomarkers or molecules (e.g., enzymes, etc.). These biomarkers can either be detected directly or used to trigger reactions that generate detectable products (e.g., enzyme/substrate reactions). Typically, directly detectable biomarkers used for functional sensing are fluorescent proteins (e.g., GFP, YFP, mCherry, etc.) [3]. In contrast, enzymatic biomarkers (e.g., β-glucuronidase, β-galactosidase, etc.) are usually observed using electroactive products generated on the reaction with corresponding substrates [4].

Electrochemical biosensing is of particular interest in sensing for precision agriculture due to its simplistic nature, and its ability to be used even under strong sunlight that usually masks optical signals. Electrochemical biosensors operating under constant voltage (chronoamperometry), demonstrate continuous drops in signal over time. Furthermore, a typical signal is in the range of nA to a few μA. This low signal amplitude, along with considerable electrical noise from the surroundings and the interfacing electronics, results in such sensors having a poor detection limit. Hence, we propose a method that uses a dual-working electrode setup and amplifies the signal, as compared to conventional electrochemical sensing, with a single working electrode. In addition, for a fixed concentration of the electroactive product, the measured current maintains its value over a relatively long time. This is in stark contrast with a single electrode system, where the current drops with time, following Cottrell’s equation. This ability of the dual-electrode system to maintain current over longer periods improves the signal level over background noise.

β-glucuronidase (GUS) has been widely used as a versatile reporter of gene expression. This enzyme is an acid hydrolase enzyme that cleaves a wide variety of β-glucuronic acids. Encoded by the uidA gene as a reporter, its use was first demonstrated in tobacco plants by Jefferson et al. [5]. The technique enables the analysis of gene expression by detecting the activity of the reporter gene (GUS). Electrochemical detection of GUS has been previously demonstrated, using the oxidation of electroactive products formed by the reaction of GUS and appropriate substrates. In particular, the detection of GUS as an enzymatic reporter of drought and heat stresses has been shown using such a methodology [2,6].

### 1.2. Amplification Using Redox Cycling

Redox cycling is an electrochemical phenomenon that was first introduced by Anderson and Reilley in 1965 [7]. Later, it was presented by Fan and Bard in their pioneering single-molecule experiment in 1995 [8]. In conventional single-electrode sensors, there is only one working electrode. In redox cycling setups, a second working electrode regenerates the molecule to its original state, “recycling” the analyte and enabling further detection at the working electrode. Each analyte molecule thus may shuttle repeatedly per unit of time between the electrodes, thus generating a current larger than a single electrode sensor. This “redox amplification” allows for an increase in the output signal. It is also likely to increase the intrinsic noise level. However, in most field deployable systems the extrinsic noise is dominant, and hence, increasing the signal may improve the limit of detection. 

The two working electrodes are referred to as the generator—where the initial oxidation occurs (e.g., R − e —» O)—and the collector—where the continuous reduction takes place (e.g., O + e —» R). They are biased individually such that the higher potential electrode generates the initial oxidation and the electrode on which the lower potential is applied reduces the analyte back to its original state, before diffusing out into the bulk of the solution. Overall, the molecule transfers charges during every successive reaction, effectively amplifying the detected current per molecule. The high redox cycling improves the signal-to-noise ratio, and the wide dynamic range allows for high sensitivity.

Interdigitated arrays (IDAs) are a favorable geometry for redox cycling due to the specific electrochemical behavior they allow [9]. The electrode couple is biased with potentials that provide sufficient over-potentials for oxidation on one electrode and reduction on the second. The species that is generated from the analyte’s redox reaction is then collected at the adjacent electrode through non-planar diffusion. The measured currents at the generator and the collector are directly associated with the diffusion characteristics between them.

The use of redox cycling has been previously demonstrated in various biological and analytical use cases [10,11,12,13,14,15,16,17,18,19,20,21], employing the ability to amplify the measured currents to detect lower concentration elements in in vitro environments, in microfluidic setups, or other laboratory settings. This study is the first to use redox cycling for in vivo plant sensing applications. An ultrasensitive detection method will open the door for low-concentration chemical detection in plants (e.g., detection of hormones like auxins and gibberellins).

Another useful effect is that whereas conventional single-electrode signals decay with time after a step function excitation, the redox-cycling apparatus yields an almost fixed current that decays very slowly. The long response time of the redox-cycling process generates a much larger output charge (number of electrons generated) per product molecule allowing working at lower bandwidth, further improving the signal-to-noise ratio. 

The generator voltammogram characteristics of a dual-working electrode (WE) mode are inherently different from that of a single-WE mode. For the oxidation reaction in a single-WE mode at low overpotential, the reaction is electrode reaction rate-limited, and the current increases exponentially with voltage. At higher overpotentials, the electrochemical reaction becomes limited by the reactants’ diffusion from the bulk to the electrode. In that case, the effective analyte concentration at the electrode surface decreases, and the current decreases with it, displaying a peak shape. In dual-WE mode, as the potential at the generator electrode approaches the redox potential, both generator and collector currents increase by the same magnitude, and an opposite sign. In the straight-forward case, as the generator potential surpassed the redox potential, the efficient cycling of the redox species between the microelectrodes of the IDA provides a constant concentration at the electrode surface, thus, as previously reported [22], producing a constant current regardless of the overpotential and displaying a typical sigmoidal shape. The hysteresis between the anodic and the cathodic current in the cyclic voltammetry (CV) curves is minimized, indicating that the quasi-steady-state is being approached.

### 1.3. Electrochemical Characterization

**Figure 1:**
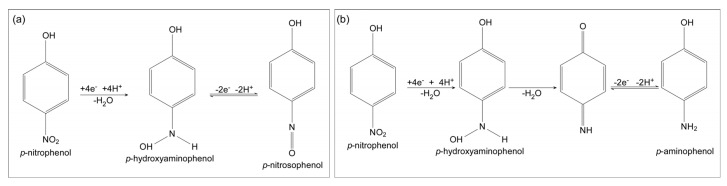
Possible p−NP electrochemical pathways that include reversible reactions as shown in the review of Tchieno and Tonle [24]: (**a**) is mentioned as mechanism II and was measured using gold electrodes. (**b**) is mentioned as mechanism III for deposition on nickel phthalocyanine electrodeposited film and glassy carbon electrodes (GCE).

The electrochemical sensor is based on the redox reactions of *p*-nitrophenol. There exists ample literature on this matter including books [23], review papers [24], and research papers showing interest in those reactions for various applications such as biosensing, electropolymerization, and water remediation [23,24,25,26,27] As mentioned in the review paper by Tchieno and Tonle [24], there are a few possible reaction pathways that are prone to occur regarding the nitro functional group, and two of them can support redox cycling (Figure 1). 

Both routes start with the *p*-nitrophenol irreversible reduction to *p*-hydroxylaminophenol as observed in the cathodic scan [23,24]. The *p*-hydroxylaminophenol molecules can be oxidized in two possible reversible reactions [24], allowing redox cycling. The first possible route (Figure 1a) includes the oxidation of *p*-hydroxylaminophenol into *p*-nitrosophenol in a reversible reaction, starting at a low positive bias [24]. The oxidation occurs on the anode, and the corresponding reduction, when there is redox cycling, occurs on the cathode [24,28,29,30]. The second possible route (Figure 1b) includes dehydration, the loss of a water molecule, and a reduction of *p*-hydroxylaminophenol into aminophenol. Similar to the previous reaction, this reaction also starts at a low positive bias. Like before, the reduction along each route may happen on the cathode while the corresponding oxidation can happen on the anode [23,24,25,26,31], allowing redox cycling. 

### 1.4. Heat Shock in Tobacco Plants

Plant gene expression can be regulated by various conditions including temperature change, light, water status, or hormone balance. It has been shown that when seedlings’ temperatures are shifted five or more degrees above optimal growing temperatures, synthesis of most normal proteins and mRNAs is repressed, and transcription and translation of a small set of “heat shock proteins” (HSPs) are initiated [32,33,34]. The response of plants to a sudden increase in the incubation temperature results in the inhibition of the synthesis of most cell proteins and increased synthesis of a relatively few proteins, “heat shock proteins” [33,35] The pattern of protein synthesis changes rapidly and dramatically when the growth temperature of seedling tissue is increased from normal (~28 °C) to heat shock (~40 °C).

In this study, we demonstrate the sensitivity of the redox-cycling sensor in detecting heat-shock treatment, and study the time-dependent behavior and compare our system to the golden-standard method (X-gluc staining). 

## 2. Materials and Methods

### 2.1. Generating the Biosensor

The plant cell suspension and plants that were used in our experiments were genetically transformed to express a reported gene by *Agrobacterium tumefaciens*-mediated transformations [36].

#### 2.1.1. Cell Culture

Tomato plant cell culture (*S. lycopersicum* line MSK8) was transformed using *A. tumefaciens* strains EHA105 harboring plasmids in the pBIS-N1 vector [37] for constitutive uidA gene expression. The cultures were grown as described by Felix et al. [38] and used 4–6 days after weekly sub-culturing in a pH 5.8 medium.

#### 2.1.2. Plant Mode

*Nicotiana tabacum* plants were genetically transformed using *Agrobacterium* strain GV3101 harboring the *uidA* gene encoding for under 35S promoter [39], or driven by heat shock inducible promoter Hsp 18.2 [40], in the pBINPLUS vector [41].

### 2.2. Electrode Chip

Thin-film interdigitated array microelectrode chips were purchased commercially (MicruX Technologies, Gijón, Spain). The electrodes are fabricated using 150 nm gold sputtering with a 50 nm titanium adhesion layer, deposited on a glass substrate, and covered by an SU-8 protective layer. The interdigitated electrodes are based on a four-electrode system. The working electrodes consist of two individually addressable arrays, arranged in a comb structure. There was a total of 30 pairs of 2 mm long, 5 μm wide electrodes with 5 μm space between them. A quasi-reference Ag/AgCl electrode was used in the planar chip. The reference electrode was made by silver electroplating followed by anodization in a chlorine salt solution [42]. 

### 2.3. Electrochemical Cell

The cell line experiments were performed in a commercial All-in-One cell (MicruX Technologies, Asturias, Spain), which contains an aluminum base with a methacrylate cover, and a PEEK batch micro-cell that assembles magnetically. Before running each experiment, a new IDA chip was taken from an individually sealed package, rinsed with isopropanol and deionized water, and dried under nitrogen flow. The chip was placed in a custom slot and sealed with an O-ring. Pogo pins were used to contact the electrodes’ contact pads and connect them to the potentiostat. 

### 2.4. Plant Model Experimental System

**Figure 2:**
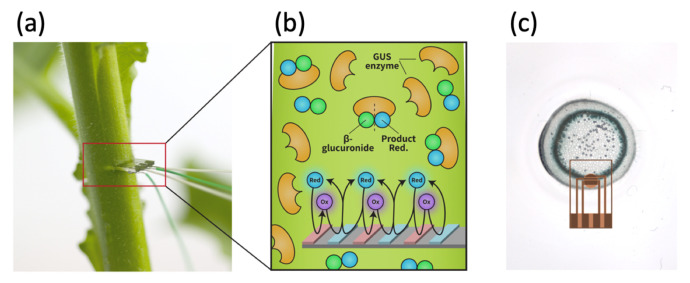
Plant model schematic. (**a**) The electrochemical chip’s connection to the plant. (**b**) Illustration of the enzymatic reaction and the electrochemical reaction. (**c**) Cross-section of the reaction surface, histochemical staining of transgenic tobacco stem cross-sections expressing GUS under the control of 35S promoter, and electrode mounting.

The plants were characterized by direct contact measurements. The IDA chip was inserted into the slit in the stem that provided continuous contact between the electrodes and the plant’s exposed cross-section (Figure 2a).

The sensing is based on the electrochemical mechanisms shown in Figure 2b. Part 1 of Figure 2b shows the enzymatic reaction: The substrate (green and blue connected circles) is cleaved by the enzyme. Part 2 of Figure 2b shows the electrochemical reaction where *p*-hydroxylaminophenol is converted to *p*-nitrophenol in a reversible reaction. The chip was positioned perpendicular to the stem—and hence the phloem tubules—facing upwards, allowing the patterned electrodes to have maximal contact with the vascular cross-section. The chip was inserted only partially into the stem, as shown in Figure 2c, such that the electrodes were in close proximity to the regions of the stem which express the enzyme. The chip was connected to the potentiostat via external soldered wires.

### 2.5. GUS Staining

Histochemical staining was done using X-gluc (5-bromo-4-chloro-3-indolyl-β-D-glucuronide) as a substrate. Stem cross-sections of transgenic tobacco plants generated by the GUS construct with 35S promoter were sliced (~0.1 mm). Once the substrate break-down occurs, blue color is observed and the enzyme distribution can be analyzed.

### 2.6. Electrochemical Characterization

All of the electrochemical characterizations were performed using a Palmsens4 potentiostat with a fixed bipotentiostat module. Every set measurement was performed once with a single-WE mode, i.e., where only one working electrode is enabled, and once with a dual-WE mode, i.e., where redox cycling is applied between two active working electrodes. The scan rate was kept at 100 mV/s. In the cyclic voltammetry for the single-WE mode, the working electrode’s potential was swept in the negative direction from 0 V to −1 V and in the positive direction to 1 V and back to −1 V. Five such cycles were performed for each measurement to ensure the signal stabilization and the second cycle in each measurement is presented in the graphs. In the dual-WE mode, the collector electrode (WE2) was biased at −1 V, and a potential sweep from −1 V to 1 V was applied to the generator electrode (WE1) for five cycles. The currents of the two working electrodes were recorded. 

MSK8 tomato cells were used for cell line experiments with and without stirring, and tobacco plants were used for direct on-plant measurements. The plant cells were suspended in Murashige and Skoog (MS) media at pH 5.8. A total of 50 μL of the cell line was inserted into the micro- chamber and 5 μL of *p-nitrophenyl β-D glucuronide* stock solution (pNPG, 0.1 M, Sigma Aldrich, Rehovot, Israel) was added. The measurement was done in constant 10 min intervals.

In the experiments on plants, 0.1 M pNPG substrate was dripped into the stem’s exposed cross-section and the reaction was immediately monitored in ten minutes intervals. The electrochemical characterization was performed using the same process as for the aforementioned experiments on cell lines.

### 2.7. Heat Shock Sensors’ Measurements

Whole plants were incubated at 37 °C for 2 h and then returned to their original growing conditions (24 °C). The GUS formation was studied by the gold-standard method (X-gluc staining) and electrochemical characterization at single and dual modes before incubation (reference measurement), after one hour, and after one, two, and three days. For X-gluc staining, leaf discs were taken from leaves close to the chip insertion area and kept at 37 °C overnight. Once the substrate is cleaved by the GUS enzyme a blue color is observed. The electrochemical measurements are conducted according to the same conditions as described before, and the maximum currents were analyzed.

## 3. Results and Discussion

The presence of the GUS enzyme was successfully detected using an improved electrochemical method based on redox cycling. This study showed the first successful implementation of in vivo redox cycling, using biological tissue as the ion diffusion medium. These studies are also the first demonstration of redox cycling for plant sensing, with in situ experiments performed on MSK8 tomato cell lines and in vivo experiments performed on tobacco plants. 

### 3.1. Cell Culture

The initial implementation of redox cycling was done in MSK8 cells transformed with uidA (GUS), under the control of constitutive expression. The GUS enzyme expressed in the cells reacts with an externally-introduced pNPG substrate. It catalyzes the breakdown of the substrate into a sugar moiety—β-glucuronide—and an electrochemical active moiety—*p*-nitrophenol. The product diffuses to the aqueous medium and then onto the microelectrode array, where a redox reaction occurs. 

**Figure 3:**
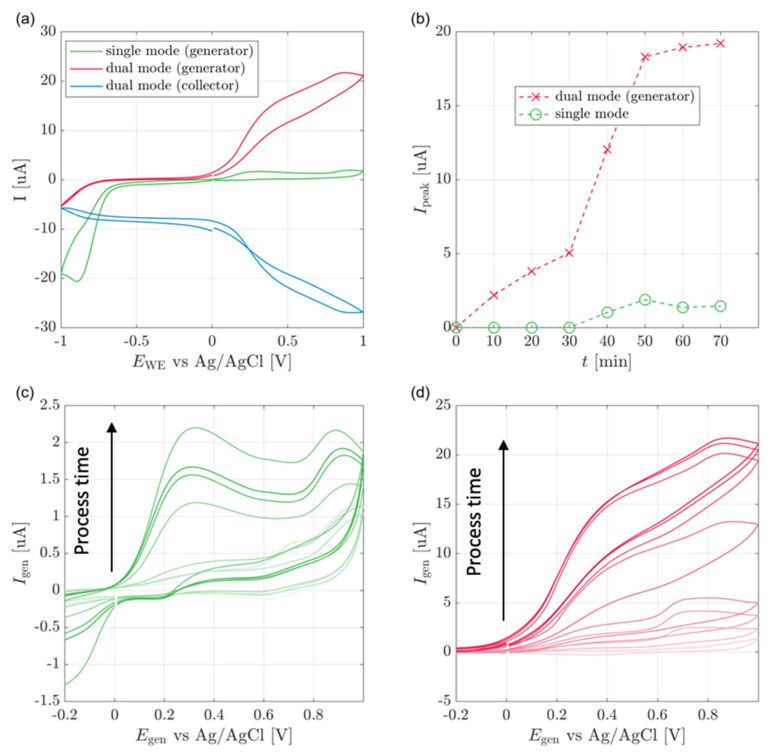
Cell line experiments (**a**) Cyclic voltammograms of MSK8 cell lines at quasi-steady-state, 70 min after the addition of pNPG substrate and (**b**) Dual and single-WE mode peak currents with respect to time from the addition of pNPG. Single-WE (**c**) and dual-WE (**d**) mode cyclic voltammograms of MSK8 cell lines at constant ten minutes intervals after the addition of pNPG. Color gradient shifts from light to dark with increasing time.

Figure 3a displays single and dual-mode voltammograms. The single mode curve shows reduction and oxidation peaks, as described in the introduction. Unlike ideal redox cycling, the dual mode presents a voltammogram where the currents increase with the potential increase. This can be explained by assuming that the redox cycling occurs between the by-products of *p*-nitrophenol reduction, the concentrations of which continuously increase, thus leading to an increase in the oxidation and reduction currents. 

Additionally, when the generator is scanned between a lower bias of −1 V and an upper bias of less than 0.2 V, the collector voltammogram shows a continuous constant reduction current resulting from the reduction of *p*-nitrophenol. At potentials between 0.2 V and 1 V, the reduction current represents a superposition of reversible and irreversible reductions.

As we see in Figure 3a, the collector current is negative in the range of −1 V to 0.2 V, probably due to the irreversible reduction of *p*-nitrophenol [24]. In contrast, at a bias higher than 0.2 V, we see an increase of both collector and generator currents due to the additional reversible oxidation reactions leading to redox cycling. This is visible since the extra generator and collector currents have the same magnitude but opposite signs.

The cell response was studied through eight cyclic voltammetry measurements starting at zero time and every ten minutes (Figure 3b). The peak current relates to the 0.2 V oxidation peak and is calculated by extrapolation of the capacitive current slope and its subtraction from the measured current values. Figure 3c shows the single-WE mode voltammogram obtained in the second cycle for each measurement. The oxidation peak current increased as a function of reaction time from 0 μA up to 2 μA. The same experiments were repeated for dual-WE mode (Figure 3d). Again, a sigmoid-shaped curve was observed at all process times, and the measured current increased over time, and was in the range of up to over 20 μA.

Aoki et al. have derived an analytical approximation for the limiting current (*I_lim_*) of redox cycling in an IDA, at dual-WE mode, by solving the two-dimensional diffusion equation in a steady-state [43].

(1) $$I_{lim}=\frac{n\cdot F\cdot A\cdot D\cdot C_{0}}{W_{g}}\cdot F\left(x\right)$$

(2) $$F\left(x\right)=\left\{0.637\;\cdot \;ln\left[2.55\;\cdot \left(1+x\right)\right]-\frac{0.19}{1+x^{2}}\right\}/x$$

Where $x\equiv \frac{W_{g}}{W_{e}}\;$. $W_{e}\mathrm{and}\;W_{g}$ are the width and the gap of the electrode, respectively. The area is $A=m\cdot l\cdot W_{e}$, where m is the number of digits in the array and l is the length of each digit. n is the number of electrons involved in the redox reaction, F is Faraday’s constant, C_0_ is the bulk concentration of the redox molecule of interest, and D is the diffusion coefficient of the same molecule. This expression is within a 2% error of the exact 2D numerical model when the ratio between the electrode-to-electrode gap and the electrode width is less than 1.7 [36]. Assuming both working electrodes have the same width, a more straightforward expression in the range of 0.1 < x < 1.2 is given by [36]:

(3) $$F\left(x\right)\approx \frac{1}{0.645\;\cdot \;x+0.36}$$

**Figure 4:**
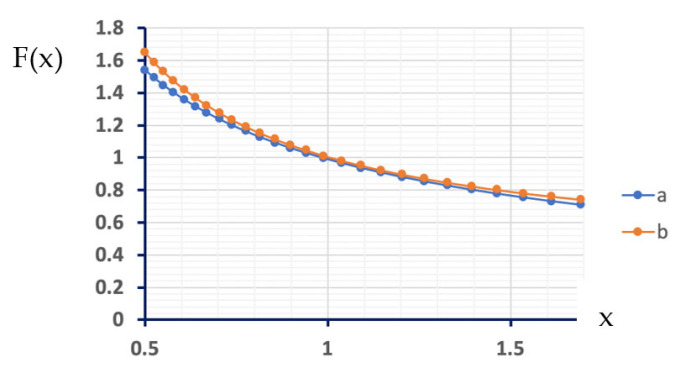
The function F(x), where x is the ratio between the electrode width to the gap between the electrodes, (a) as given by equation 2 and (b) by Equation (3) [44].

Both functions are shown in Figure 4 in the range of 0.5–1.5, which is near the experimental setup used in the work.

The following calculation assumes that the diffusion coefficients of the molecules involved in the redox cycling are similar to that of *p*-nitrophenol in water at room temperature (9.19×10−6cm2sec) [45]. In addition, we assume that at a quasi-steady state, the substrate pNPG has reacted completely to form *p*-nitrophenol. Using those assumptions, the limiting current, as found by equations 1–3, is approximately 55 μA. However, the experimental limiting current of the reaction was 11 μA, which is lower than the theoretical value, although on the same scale. 

The single and the dual-WE mode measurements show an increase in peak currents over process time. The time dependence is determined by the sum of all characteristic time constants relating to the enzymatic reaction (governed by Michaelis–Menten kinetics), diffusion of analyte in the cell, and redox cycling attainment of quasi-steady state, governed by equations 2–4, respectively. 

As presented in Figure 3b, in dual-WE mode, the reaction progress is characterized by a mild increase and then a sharp increase at forty minutes of process time. After that, the max currents continuously increase subtly and stabilize at a quasi-steady-state around t = 60 min. The single-WE mode does not show any signal before t = 40 min. At that point, a sharp increase followed by stabilization of the current is observed, similar to observations in dual-WE mode. The kinetics of enzymatic reactions under the given boundary and initial conditions can explain this behavior. The kinetics of the enzymatic cleaving can be described by the Michaelis–Menten model:

(4) $$S+E\;\;\;{\;}_{\underset{k_{-1}}{\leftarrow }}^{\overset{\;\;\;k_{1}}{\to }}\;ES\;\overset{k_{2}}{\to }\;E+P$$

where S, E, ES, and P are the concentrations of the substrate, enzyme, enzyme-substrate complex, and product, respectively, near the electrode.

(5) $$\frac{d\left[P\right]}{dt}=\frac{V_{max}\left[S\right]}{K_{m}+\left[S\right]}$$

In our case, we assume that the substrate’s concentration was high enough, i.e., $\left[S\right]\gg K_{m}$, the product’s concentration is expected to increase linearly with time. Following its generation, the product may diffuse towards the electrode and be oxidized, or flow away from the electrodes. 

Two common measurement modes of such devices were used: chronoamperometry and cyclic voltammetry. In the case of chronoamperometry, the simple 1D model for the diffusion current is (Cottrell’s model):

(6) $$I_{D1}\approx nFAD\;\cdot \;\frac{\left[P\right]}{L_{D}}\;\;where\;L_{D}=\sqrt{\pi Dt}$$

where n is the number of electrons per ion (molecule) reacting, and D is the product diffusion coefficient [cm^2^/V·sec], A is the electrode area [cm^2^], F is Faraday’s constant [Cb/mol], and t [sec] is the time from the addition of the substrate. [P] is the product concentration [cm^−3^].

According to this simplified model, the current should be inversely proportional to the square root of time since the addition of substrate. For very short time periods, the current is bounded by the electrode oxidation (reduction) rate. Assuming $k_{s}\;$ is the electrode reaction rate constant [cm/sec], the time when the diffusion becomes the rate-limiting factor is tDF≈Dks2. This time constant is usually minimal and is generally neglected, unless in cases of very fast measurements. 

Experiments show a delay in the range of a few minutes to a few tens of minutes. A simple approximation of the delay assumes a diffusion-limited time constant tD≡L22D, where L is the characteristic dimension of the electrochemical cell, usually taken as the distance between the working and auxiliary electrodes. 

In the case of redox amplification, the product is oxidized on one electrode, and the electrodes between the oxidized species defuse to the other electrode, where they are reduced and converted back to the product that diffuses to the other electrode and vice versa. In this case, when the distance between the electrodes is smaller than the diffusion length, L_D_, the total current is given by Aoki’s model [43], Equations (1)–(3). That gap between the electrodes can be reduced by a few orders of magnitude, thus increasing the current as long as it is less than the electrode reaction rate-limited current. Note that this electrode reaction rate is controlled by the overpotential and can be increased at will.

Assuming the Randles–Ševčík equation at room temperature, the peak current of a single mode CV is:

(7) $$i_{p}=2.69\times 10^{5}\;\cdot \;n^{\frac{3}{2}}\;\cdot \;A\;\cdot \;D^{\frac{1}{2}}\;\cdot \;v^{\frac{1}{2}}\;\cdot \;\left[P\right]$$

where the concentration of [P] is in molar units, and v is the scan speed, whereas in the case of redox cycling, the current reaches a maximum and does not drop beyond the maximum. The maximum current is the same as in the case of chronoamperometry and it is independent of the sweep speed. In this case, the amplification achieved in our case:

(8) $$Amplification=\frac{i_{R}}{i_{p}}\approx \frac{\left[\frac{nFAD\left[P\right]}{W_{g}}\right]\times F\left(x\right)}{2.69\times 10^{5}\;\cdot \;n^{\frac{3}{2}}\cdot A\cdot D^{\frac{1}{2}}\cdot \;v^{\frac{1}{2}}\cdot \left[P\right]}=3.59\times {\left(\frac{D}{nv}\right)}^{\frac{1}{2}}\cdot \;\frac{F\left(x\right)}{W_{g}}\;$$

This 1D model indicates that the smaller the distance between the electrode, the larger the amplification. In our case, the calculated amplification is 46.2, which gives an upper limit to the possible amplification without any losses, which is in the same order of magnitude as the measured amplification of 10–12. Note that the gain is inversely proportional to the gap between the electrodes. Technology today allows for the manufacturing of electrodes with a few nanometers spacing. Hence the gain can be a few orders larger than what has been found experimentally and reported in this work. 

The time required for the signal to rise noticeably above the noise level, after application of the substrate is referred to as the delay. In the absence of flow, the expected delay for a single electrode case was calculated to be in the range of tens to hundreds of minutes, consistent with the 40 min delay that was observed.

The sensor’s detection time significantly improved when applying dual-WE compared to single WE. In single-WE mode measurements, the signal was observed only after 40 min of process time. In contrast, in dual-WE mode measurements, the signal was observed starting from the first measurement after 10 min of process time. This improvement in detection capabilities corresponds to the detection of lower analyte concentrations in dual electrode setups, allowing for the sensing of biomarkers that were not been detectable before.

Finally, to establish that none of the components other than *p*-nitrophenol are electrochemically active, cyclic voltammetry was performed using three different reference solutions: (a) medium (which was used for cell growth and experimentation), (b) medium with commercially available GUS, and (c) medium with commercially available *p*-nitrophenol (Supplementary Materials Figure S2). One single peak was observed corresponding to *p*-nitrophenol, and none of the other components exhibited electrochemical activity.

### 3.2. Plant Stem-Mounted Sensor

After verifying the efficacy of redox cycling amplification in MSK8 cell lines, the effectiveness of this technique was studied in vivo using whole plants. Redox cycling is inherently dependent on electrolyte and medium parameters. Therefore, the challenge of using this novel technique in vivo lies in the relative lack of knowledge about the diffusion characteristics in tobacco stems and the non-uniform composition of the plant’s vasculature.

X-gluc staining of the tobacco plant stem expressing the uidA gene under the control of constitutive (35S) promoter is presented in Figure 2c. In the transverse section of the tobacco stem, inner phloem tissues and the transporting sieve tubes [46] were specifically stained with X-gluc, as previously shown by Saito et al. [47]. 

The electrochemical chip was inserted into the stem of a tobacco plant that constitutively expresses the uidA gene, after forming an initial cut in the cortex (see Figure 1a,b). In order to provide close contact with the regions of the stem that express the enzyme, the chip was inserted only partially into the stem, as shown in Figure 2c. During the normal state of the plant, the phloem transports glucose from the leaves to the rest of the plant, where the flow occurs in the capillaries as a result of concentration gradients in the different parts of the plant. When the stem is cut, small droplets continue forming on the cut’s surface, and the liquid comes into contact with the electrodes. The chip was positioned and inserted with only approximate knowledge of the location of the phloem, and without exact guidance. Hence, the position of the chip is likely to have changed slightly between different measurements. It was ensured that the cut and the chip affected less than 20% of the stem cross-section, thus not risking the viability of the plant. The positioning of the electrode chip can lead to two scenarios:

Scenario a: When the electrodes are placed directly in the active expression area, i.e., near the exposed phloem vessels, the substrate reacts with the enzyme in the vessels, and the product can immediately undergo the electrochemical reaction on the electrodes.

Scenario b: When the electrodes are not in direct contact with the phloem, the substrate reacts with the enzymes in the vessels, and the product must diffuse to the electrodes to undergo the electrochemical reaction. The diffusion of the product likely occurs through the exposed surfaces. The distance between the phloem ring and the electrodes affects the time delay due to the diffusion, and effectively also the concentration at the electrode surface.

**Figure 5:**
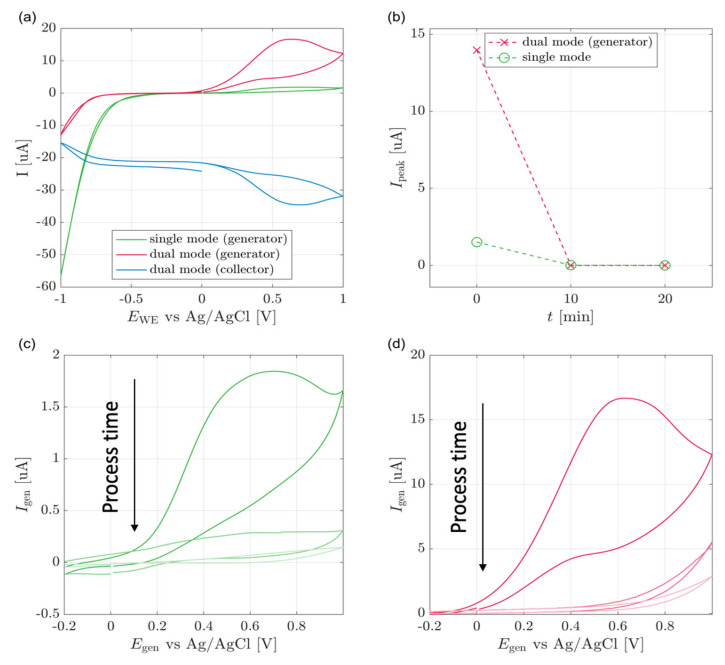
Experiments on whole plants. (**a**) Cyclic voltammograms at quasi-steady-state, at the time of the injection of pNPG substrate (**b**) Dual and single-WE mode peak currents with respect to time from the addition of pNPG. Single-WE (**c**) and dual-WE (**d**) mode cyclic voltammograms at constant ten minutes intervals after the addition of pNPG. Color gradient shifts from light to dark with increasing time.

Figure 5a,b shows the generator current in single and dual-WE mode CV at the time of injection and after ten and twenty minutes from the injection. Immediately after the injection of the substrate (t = 0), the CV shows a clear p-nitrophenol peak in both measurement modes. From that we learn that the complete process has occurred: the substrate (pNPG) has diffused into the stem; reached cells that express the enzyme (GUS), and has been cleaved to form the electrochemically active product (*p*-nitrophenol), and the product has diffused to the electrodes. The peak current obtained at dual-WE mode was 14 mA and the current obtained at single-WE mode was one order of magnitude smaller at 1.5 mA.

The subsequent measurements presented in Figure 5c,d, taken after ten and twenty minutes from the injection, showed no peaks. From this, we conclude that the analyte concentration at the electrode surface decreased from a noticeable concentration to a negligible concentration within ten minutes.

This finding is different than that of the in situ cell lines experiment. The fundamental difference between the two experimental systems is that in closed systems, such as the reaction chambers used for cell line experiments, the analyte is confined to the section, and its total amount is unchanged. In contrast, the stem is not a closed system. The xylem and the phloem are vessels that transfer fluids in the plant. We hypothesize that part of the enzymatic product is transported away from the electrode regime and, therefore, its effective concentration at the electrode surface decreases.

In addition, it was observed that in plant-based setups, the delay to the response current is minor, in the range of a few seconds. The analyte flow in the stem provides fast mass transfer toward the electrodes. Redox cycling amplification was observed for all aforementioned experimental setups, with over ten-fold amplification compared to traditional electrochemical methods. 

**Figure 6:**
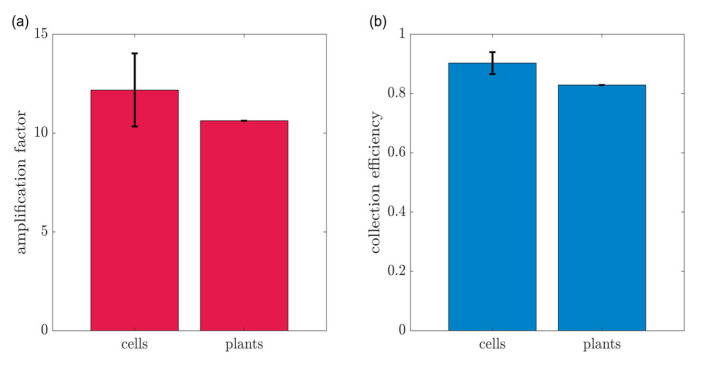
(**a**) Amplification factors and (**b**) collection efficiencies measured in experiments with a summary of cell lines and plant model.

The amplification was characterized by its amplification factor and the collection efficiency as presented in Figure 6. 

The amplification factor is the ratio between the dual-WE mode generator current and the single-WE mode current. This parameter also relates to the average number of oxidation-reduction cycles that every analyte molecule goes through, as one electron’s current is added to the measured current in each redox cycle. The amplification factor is presented in Figure 6, where the measured values range between 10 and 12. The collection efficiency for dual-WE mode measurements is defined as the ratio between the currents in the collector and the generator electrodes. A 100% collection efficiency indicates complete cycling, where all molecules are cycled infinitely. In all of the experiments conducted in this study, very high collection efficiencies of 83% to 92% were observed. This implies excellent cycling and, therefore, high amplification. The collection efficiency observed in experiments with cell lines was more than 90%, whereas in experiments with plants reached 83%. The difference is most likely driven by the fact that the cell lines are a closed system where the molecules are enclosed, contrary to the plant where the molecules flow through the stem and are less likely to reach the collector.

### 3.3. Heat Shock Sensor

**Figure 7:**
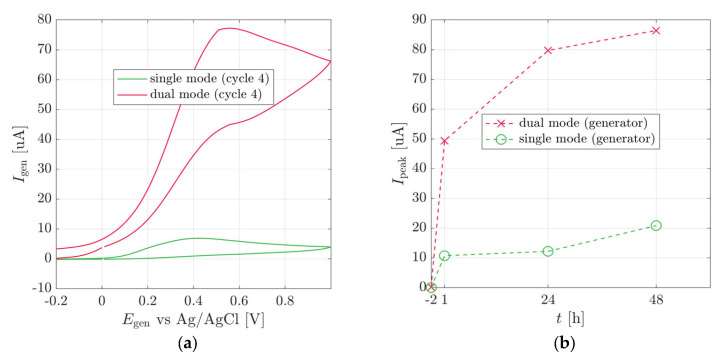
Heat-shock response: (**a**) typical cyclic voltammetry of plant stem mounted electrodes on heat shock response plant sensor after 24 h (Scan speed 100 mV/s) and (**b**) the net current response (Peak current minus the baseline displacement current) to the heat shock as a function of time after applying the stress.

To demonstrate our concept as a functional sensor, we use a heat shock promoter HSP18.2 to control the expression of GUS. Transgenic tobacco plants harboring the heat sensor were subjected to 37 °C for two hours and then returned to room temperature. The electrochemical signal was measured similarly to that described in the previous part, and the sensing paradigm was the same (Figure 7). 

CV was measured on the stem-mounted sensor, before and after the heat shock stimulus. Figure 7 presents the maximum currents obtained by the CVs in single and dual modes. Before the heat shock, no electrochemical signal was observed. After the heat shock, the signal increased gradually, starting one hour after the stimulus. The dual-electrode redox cycling signal was about nine times higher than the single mode, demonstrating the amplification effect of this mode. The redox cycling current enables a high signal-to-noise ratio due to the higher signal at dual mode.

**Figure 8:**
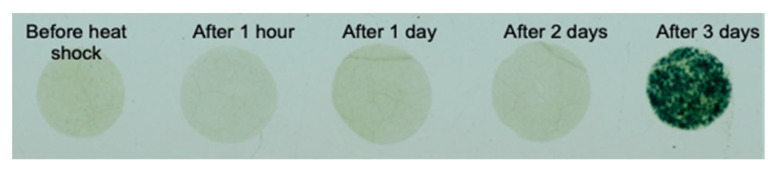
Staining experiment for heat shock sensing plants: (Left to right) before heat shock, after 1 h, 1, 2, and 3 days after applying the stress.

Figure 8 presents the gold-standard method (X-gluc staining) described in the experimental section. Using X-gluc, the heat shock effect was observed three days after applying the stress, significantly longer than the one-hour detection time by electrochemical methods.

## 4. Summary and Conclusions

This work presents a plant-based sensing system, combining functional biosensing with a redox cycling-based electrochemical transducer. The sensor was implemented in situ in MSK8 tomato cells and in vivo on tobacco plants. Specifically, the cells were designed to express GUS constitutively, and the plants were designed to express it either constitutively or as a response to heat shock. In the two models, cyclic voltammetry was performed. One order of magnitude amplification was measured in dual-electrode setups with redox cycling, as compared to standard electrochemical measurements. 

The results of the presented studies have shown enhanced sensitivity of the redox cycling-based method compared to standard electrochemical methods. The collection efficiency of the dual-WE setups was in the range of 83–92%, and the amplification factor was between 10 and 12. In the cell suspension, the enhanced sensitivity due to the amplified electrochemical current allowed for very early detection of enzymatic activity using low concentrations of biomarkers. The enzymatic product was successfully detected in 10 min using the dual-electrode setup, significantly sooner than 40 min for the conventional single-electrode setup. In the whole plant-based heat shock sensor, the electrochemical sensor’s signal was detected after one hour, compared to three days with the gold-standard method. The redox cycling provided a nine-fold amplification of the measured signal. It is possible to further increase the amplification by decreasing the distance between adjacent electrodes. 

It has been established by previous works that GUS expression in plants can be detected through the plants’ leaves [2,6] This study shows that the stem is a good candidate for monitoring enzymatic expression. As many plants undergo leaf-shedding during winter, monitoring the enzyme expression through the stem presents a practical advantage. In addition, measurement through the stem is preferable from an engineering perspective. The insertion of the microchip into a slit in the stem provides durable and steady contact between the electrodes and the active surface of the plant. Moreover, the transport features of the media play an essential role in determining the dynamics of the measurements. The signal of the “open system” of the plant is faster to rise and also quicker to decrease. Therefore, measuring electrochemical signals in the stem provides better time resolution than measuring the same in leaves, distinguishing between different events throughout the measurement.

This effect can be used to monitor hydration, nutrient deficiency, photosynthetic efficacy, etc. The challenge in sensing through the stem lies in the inherent localization of the enzymatic expression—which is limited to specific cells—in contrast; the entire mesophyll exhibits enzymatic expression in the leaves.

The proposed method may have some practical concerns. For example, inserting a chip into the stem may affect the plant; hence future designs should minimize the damage to the plant structure. Further work is needed to assess the signal’s long-term stability and reliability. Electrochemical bio-sensing is relatively simple, and the device described here is no exception. The mounted IDA uses standard low-cost measurement equipment, which can be acquired “off-the-shelf”. The sensors can be implemented as part of an active agricultural setup, providing accurate, direct, and immediate signals for any stress the plant is experiencing. The initial proof-of-concept studies were designed to express GUS constitutively; we demonstrated that it could report heat shock stress.

The amplification of electrochemical currents can also allow the detection of other biomolecules in plants in low concentrations. Detection of such biological compounds, including auxins and gibberellins, is of great importance in detecting the status of plants’ health and well-being. In addition, biological functional signals thus measured have the potential to have high accuracy and low delay, thus serving as reliable data for precision agriculture systems.

## Data Availability

Not applicable.

## Supplementary Materials

The following supporting information can be downloaded at: https://www.mdpi.com/article/10.3390/bios13020219/s1, Figure S1: Experiments on cell lines performed with stirring. (a) Cyclic voltammograms of MSK8 cell lines at quasi-steady-state, 20 minutes after addition of pNPG sub-strate (b) Dual and single-WE mode peak currents with respect to time from the addition of pNPG. Single-WE (c) and dual-WE (d) mode cyclic voltammograms of MSK8 cell lines at constant ten minutes intervals after the addition of pNPG. Color gradient shifts from light to dark with in-creasing time; Figure S2: Single and dual-WE mode cyclic voltammetry of the cell growth media, with additions of GUS enzyme and commercially available p-nitrophenol. Under single-WE mode, the WE is scanned from -1 V to 1 V at a 100mV/ sec scan rate, and under the dual-WE mode, WE2 is set constant at -1 V and WE1 is scanned from −1 V to 1 V.
