# Supplementary material for: In Vivo Plant Bio-Electrochemical Sensor Using Redox Cycling
Source: Biosensors (Basel). 2023 Feb 2;13(2):219. doi: 10.3390/bios13020219 (PMC9953906; doi:10.3390/bios13020219)
Supplement: Supplementary file 1 [file biosensors-13-00219-s001.zip › biosensors-2159087-supplementary.pdf]

biosensors

MDPI

Article

Supplementary Materials:

1S: Experimental section:

In the experiments with stirring that are shortly discussed, and presented in Figure 1, 0.5 ml substrate was added to a beaker with 5 ml of cells solution in MS media and stirred using a mild magnetic stirring. The electrochemical characterization was performed using the same process as for the aforementioned experiment without stirring.

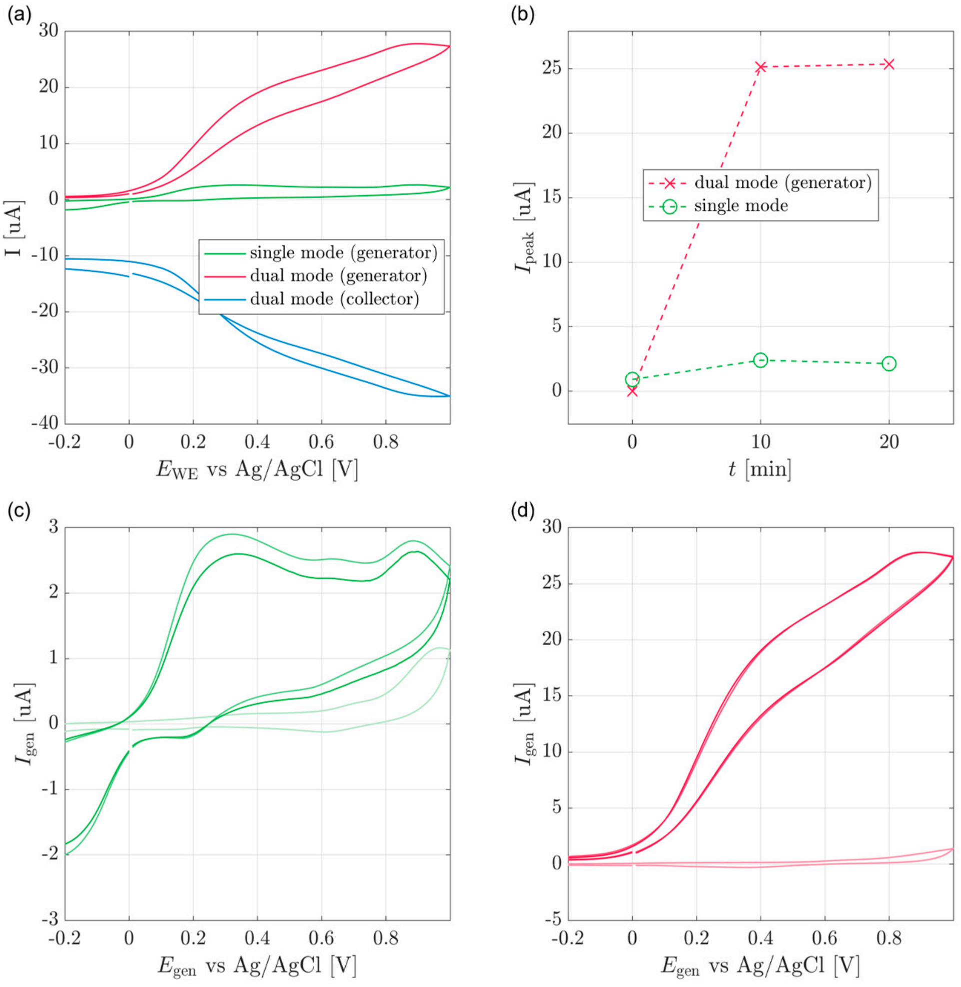

Figure S1: Experiments on cell lines performed with stirring. (a) Cyclic voltammograms of MSK8 cell lines at quasi-steady-state, 20 minutes after addition of pNPG substrate (b) Dual and single-WE mode peak currents with respect to time from the addition of pNPG. Single-WE (c) and dual-WE (d) mode cyclic voltammograms of MSK8 cell lines at constant ten minutes intervals after the addition of pNPG. Color gradient shifts from light to dark with increasing time.

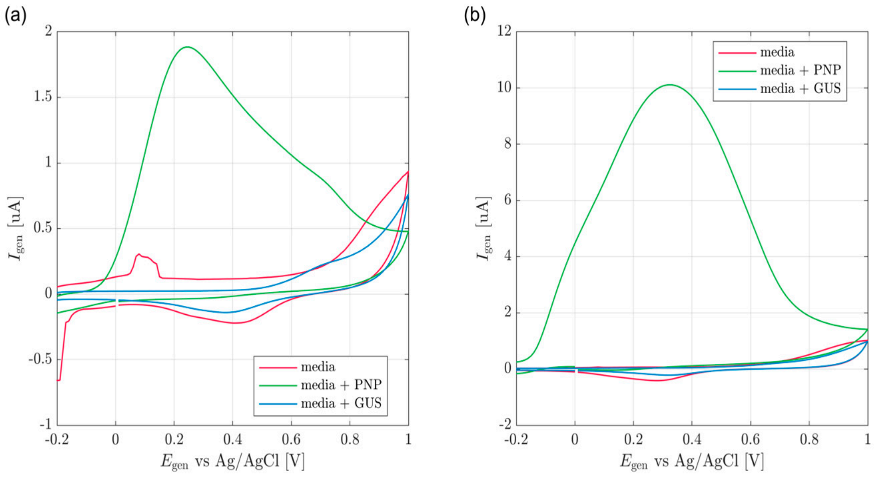

Figure S2: Single and dual-WE mode cyclic voltammetry of the cell growth media, with additions of GUS enzyme and commercially available *p*-nitrophenol. Under single-WE mode, the WE is scanned from -1 V to 1 V at a 100mV/ sec scan rate, and under the dual-WE mode, WE2 is set constant at -1 V and WE1 is scanned from -1 V to 1 V.
